# MIL-53(Al) catalytic synthesis of poly(ethylene terephthalate)

**DOI:** 10.55730/1300-0527.3434

**Published:** 2022-04-26

**Authors:** Ji WANG, Qingyin WANG, Gongying WANG

**Affiliations:** 1 Chengdu Institute of Organic Chemistry, Chinese Academy of Sciences, Chengdu, China; 2 National Engineering Laboratory for VOCs Pollution Control Material & Technology, University of Chinese Academy of Sciences, Beijing, China

**Keywords:** Green catalysis, metal-organic frameworks, poly(ethylene terephthalate), aluminum-based catalyst

## Abstract

Poly(ethylene terephthalate) (PET) is one of the world’s five major engineering plastics and is widely used in various fields. At present, the main catalysts used in the synthesis of PET are antimony, titanium, and aluminum metal compounds. Among them, antimony-based catalysts are poisonous and the titanium-based catalyst products are relatively yellow in hue. The aluminum-based catalyst has the advantages of low price and environmental friendliness, but current research shows that the organoaluminum catalyst has the problem of hydrolysis, and MIL-53 (Al) has good stability and will not affect the environment, so we add a catalyst before the esterification reaction, and uses thermogravimetric analysis (TGA), X-ray diffraction (XRD), scanning electron microscope (SEM), and N_2_ low-temperature physical adsorption characterizes the thermal stability and structure of MIL-53(Al). At the same time, the effects of different content, polycondensation time, before and after activation and polycondensation temperature on the properties of PET were investigated. The research results show that when the molar content of catalyst is 0.05% and the reaction temperature is 280 °C for 150 min, the product obtained is relatively excellent. The catalytic activity has almost no effect before and after activation, indicating that the polycondensation reaction is carried out on the surface of the catalyst. Therefore, MIL-53 (Al) has great potential in PET industrial catalysis.

## 1. Introduction

Poly(ethylene terephthalate) (PET) is one of the world’s five major engineering plastics. It is widely used in the production of fibers, films, and various types of containers, and it is used in all areas of people’s lives [1–4]. The global demand for PET is still increasing year by year at an annual rate of 8%. It is estimated that the production of PET in 2022 will exceed 25 million tons. In the production process of PET, the catalyst plays a very important role. The catalyst can not only affect the molecular weight of the polyester and the polycondensation reaction rate, but also has a significant impact on the thermal stability and chromaticity of the polyester. Therefore, it is necessary to comprehensively consider several aspects such as its catalytic activity, environmental protection performance and its thermal stability in the selection of polyester catalyst. At present, the most used polyester polycondensation catalyst in the industry is an antimony-based catalyst, and the resulting product has a uniform molecular weight, few by-products and low price. But antimony ions are harmful to the human body and can be separated out from the product. Products containing antimony ions will also diffuse into the environment during the spinning process and cause environmental pollution. This limits the application of antimony-based catalysts in the PET polymerization process. Therefore, looking for environmentally friendly and nontoxic and harmless catalysts is the current research focus [1,5–8].

Titanium-based catalysts are environment-friendly catalysts and have well catalytic activity. Typical representatives of titanium-based catalysts, such as tetrabutyl titanate, isopropyl titanate, simple chelates, or inorganic salts, have low selectivity for the polycondensation and degradation reactions in the polyester polycondensation process. In addition, it is easy to hydrolyze to form a titanium oxide compound during the esterification process, which not only greatly reduces the catalytic activity but also increases the haze of the polyester. Therefore, the synthetic polyester has a yellowish color and a high carboxyl end group value. Isamu Shigemoto et al. [9] used quantum chemistry to calculate the effects of 19 titanium-based catalysts on the polycondensation and depolymerization processes of PET. In addition, calculations indicate that catalysts with aromatic rings will have a negative impact on the color of the product, but if sugar alcohol molecules are used as the second ligand, the catalyst can be more selective. The addition of sugar alcohol ligand can increase the activity of the catalyst for the polycondensation reaction and reduce the thermal degradation activity, while also improving the color of the polymer. Therefore, a reasonably designed titanium catalyst with high selectivity and hydrolysis resistance has been widely concerned.

Aluminum-based catalysts and antimony-based catalysts have the same active sites as trivalent metals, but the former is low in price and light metal that has little impact on the environment and the human body. Therefore, the research of aluminum-based catalysts in the polycondensation reaction of PET has also attracted much attention. At present, the application of aluminum-based catalysts has shown good prospects, and it is also an inevitable trend to replace antimony and titanium-based catalysts. In previous reports, Bin Xiao et al. compared the performance of a large number of aluminum-based catalysts and proposed three reasons to explain the difference in activity between the catalysts [10]. Among them, the activity of ethylene glycol aluminum is the best and is close to that of ethylene glycol antimony. Although it has excellent catalytic performance, it is unstable in water, which limits its application in industry. Qinghui Lin et al. [11] supported AlO(OH) on the attapulgite whose surface is full of hydroxyl groups and pointed out that hydroxyl and AlO(OH) have a synergistic catalytic effect. The intrinsic viscosity of the obtained polyester is 0.661 dL/g. At present, aluminum-based catalysts have the problem of hydrolysis, so exploring stable aluminum-based catalysts with excellent catalytic performance is the direction of future research.

Since the 1990s, porous materials of metal-organic framework (MOF) materials self-assembled by coordination of metal nodes and organic ligands have been synthesized and studied in large quantities [12–21]. Different from traditional porous materials, MOF has designability and pore controllability. It can be synthesized by selecting different metal ions or metal clusters and different organic ligands, and the pore size can be adjusted by adjusting the size of organic ligands. Therefore, MOF material is one of the ideal materials for catalysis [22–30]. MIL-53(Al) is a widely used aluminum-based MOF material. It can exist stably at a high temperature of 500 °C in the presence of oxygen, can be synthesized by a green method, and is one of the commercial MOFs [31–33]. Madhan Vinu et al. [34] synthesized five Al-based MOF materials as catalysts for selective sulfoxidation reactions, and the results showed that MIL-53(Al) exhibited excellent catalytic effects at different temperatures. Ehsan Rahman et al. [35] used MIL-53(Al) and MIL-53(Al-Li) to catalyze the alkylation of benzene reaction for the first time, and the insertion of Li atoms improved the yield of the main product because Li atoms can adjust the acidic sites of the catalyst to achieve the optimal catalytic effect.

Unfortunately, the application of MIL-53(Al) as a catalyst to the PET synthesis process has not been reported. In this study, unactivated MIL-53 *as* (Al) and activated MIL-53 *lt* (Al) were used as PET polycondensation catalysts for the first time. Compared to other Al-based catalysts, MIL-53(Al) has excellent water stability so it can be added before esterification without hydrolysis. The effects of the two catalysts on the viscosity of PET at different polymerization temperatures, different amounts of catalysts, and different polymerization times were investigated. The results show that there is almost no difference in the catalytic performance of the two catalysts. It is proved that the reaction is carried out on the surface of the catalyst.

## 2. Materials and methods

### 2.1. Chemicals and instruments

Benzene 1,4-dicarboxylic acid (BDC, 99%) was purchased from Aladdin Biochemical Technology Co. Ltd. Ethylene glycol (98%), N,N-dimethylformamide (99.5%), methanol (99.9%), ethanol (99.8%) and acetone(99%) were supplied by Chengdu Kelong Chemical Company. Aluminum nitrate nonahydrate (99.99% metals basis) was obtained from McLin Biochemical Technology Company. Phenol (Jiangsu Yonghua Chemical Company, 99%), 1,1,2,2-tetrachloroathane (Jiangsu Qiangsheng Chemical Company, 99%) were used as received. All the reagents were purchased and not further purified.

The powder XRD pattern was collected on a PANanalytical X’Pert Pro diffractometer operated at 40 kV and 40 mA and using Cu Kα radiation in the 2θ angular range of 3–80°.

The simulated powder diffraction pattern of the crystal is given by the software Mercury.

The Brunauer-Emmett-Teller (BET) surface calculations were determined by N_2_ adsorption-desorption isotherms via a Micromeritics Tristar 3020. N_2_-isotherms were measured at 77 K. Degassing of the samples was conducted under vacuum at 423 K for 12 h.

Thermogravimetric analysis (TGA) was performed under a nitrogen atmosphere using NETZSCH TG209F1 equipment with a heating rate of 10 °C/min.

The electron microscope used in the experiment was produced by the German company ZEISS and the model was Gemini SEM300.

### 2.2. Synthesis of MIL-53(Al)

MIL-53 was synthesized according to a modified synthesis scheme [36]. Briefly, Al(NO_3_)_3_·9H_2_O (0.667 g, 6 mmol) and benzene 1,4-dicarboxylic acid (0.498 g, 3 mmol) were suspended in 5 mL deionized water and stirred at room temperature for 30 min. The final suspending solution was put into a Teflon-lined stainless-steel container (25 mL) and heated at 220 °C for 3 days. The container was then allowed to cool to room temperature and centrifuged to get a white powder. The obtained white powder was washed with N,N-dimethylformamide and acetone three times to remove unreacted benzene 1,4-dicarboxylic acid. Then it was dried at 150 °C for 12 h, and the product obtained was MIL-53 *as* (Al) which was calcined at 330 °C for 3 days to get MIL-53 *lt* (Al).

### 2.3. Synthesis of PET

A certain proportion of benzene 1,4-dicarboxylic acid (6 mol), MIL-53, and ethylene glycol (7.8 mol) was added into a 5 L polyester synthesis device equipped with a process tower, cooling tower, and a vacuum system. Slowly stir the reactants, and then replace the air with nitrogen two to three times, fill with nitrogen and pressurize to 200 kPa, and then heat the reaction system to 260 °C, and the esterification reaction ends when the water yield reached 95% of the theoretical value. Then the vacuum system of the device was opened, and the pressure was reduced to below 100 Pa within 1 h. After the vacuum was established, the polycondensation reaction was timed. When it is completed, 150 kPa nitrogen was filled again to press the product, and the product was cut and dried for preservation.

### 2.4. Intrinsic viscosity (IV) measurements

0.125 g of PET was dissolved in 25 mL phenol:tetrachloroethane (m:m = 50:50) solution, and use an Ubbelohde viscometer to measure the intrinsic viscosity in a glass constant temperature water bath at 25 °C. It is in accordance with formulas (1) and (2) to perform calculations:


(1) $$\eta_{sp}=\frac{t_{1}-t_{0}}{t_{0}}$$

(2) $$[\eta ]=\frac{\sqrt{1+1.4\eta_{sp}}-1}{0.7c}$$

The viscosity-average relative molecular mass M_η_ is related to the IV by the following equation (3): [11]


(3) $$[\eta ]=2.1\times 10^{-4}M_{\eta }^{0.82}$$

## 3. Results and discussion

### 3.1. Characterization of MIL-53(Al)

**Figure 1:**
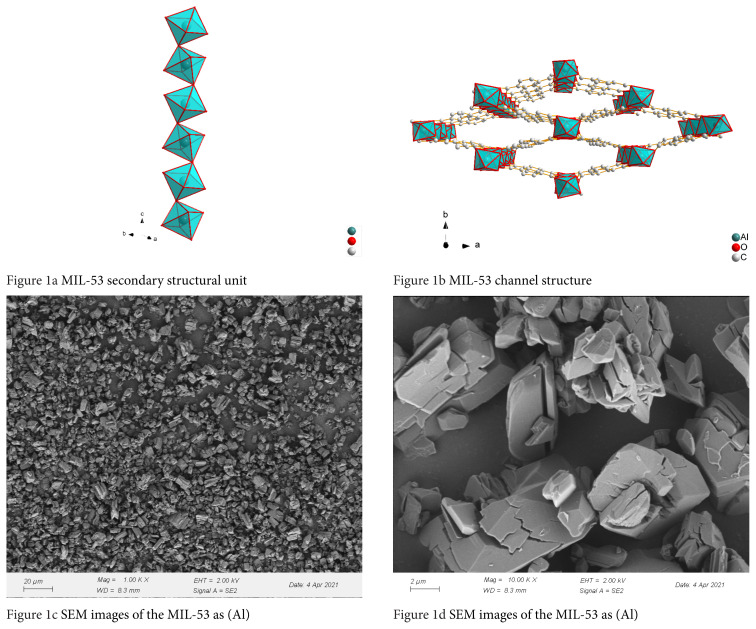
MIL-53 secondary structural unit (a) MIL-53 channel structure (b) SEM images of the MIL-53 as (Al) (c)(d). Figure 1a MIL-53 secondary structural unit Figure 1b MIL-53 channel structure Figure 1c SEM images of the MIL-53 as (Al) Figure 1d SEM images of the MIL-53 as (Al)

The structure of MIL-53(Al) is shown in Figure 1a. The aluminum atom and six oxygen atoms form a secondary structural unit of AlO_4_(OH)_2_. Adjacent secondary structural units are connected in the c-axis direction by sharing an oxygen atom to form a chain structure. Terephthalic acid connects all the secondary structural units to form a three-dimensional network structure as shown in Figure 1b. SEM images of powder samples synthesized are shown in Figures 1c and 1d. It can be seen that the crystal size of MIL-53 *as* (Al) is between 2–10 μm, which is close to that reported in the literature [37].

**Figure 2:**
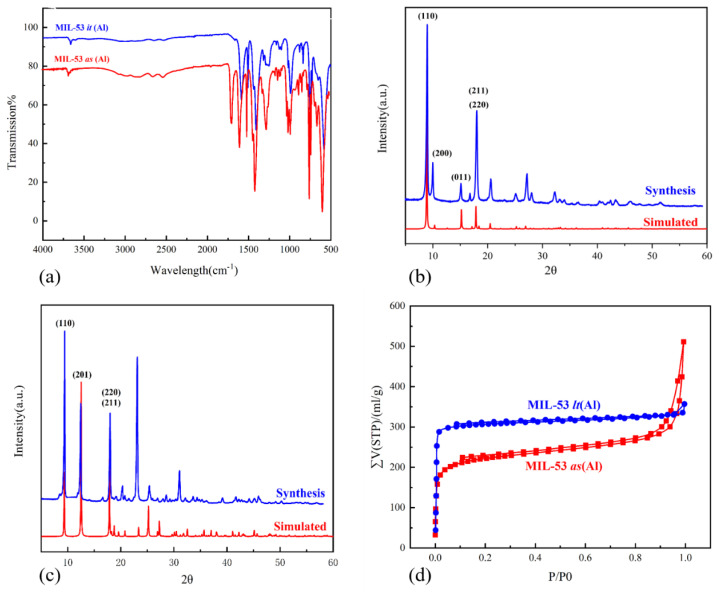
IR spectra of MIL-53 as (Al) and MIL-53 lt (Al) (a) XRD pattern of MIL-53 as (Al) (b) XRD pattern of MIL-53 lt (Al) (c) N_2_ adsorption and desorption isotherm of MIL-53 as (Al) and MIL-53 lt (Al) (d).

The IR spectra of MIL-53 as (Al) are shown in Figure 2a. There are two absorption bands at 1406 cm^−^^1^ and 1443 cm^−^^1^. These can be assigned to -COO symmetric stretching, whereas bands at 1507 cm^−^^1^ and 1585 cm^−^^1^ can be attributed to -COO asymmetric stretching. The -COO group coordinated with aluminum was proved by the appearance of these absorption bands. The stretching vibration peak of the -OH group attached to the aluminum atom was found between 3500 cm^−^^1^ and 3600 cm^−^^1^. It is worth noting that the additional absorption peak at 1695 cm^−^^1^, which is the characteristic absorption band of the free carboxylic acid, indicates the presence of unreacted terephthalic acid in the pores. Except for the absorption band at 1695 cm^−^^1^, the IR spectrum of MIL-53 lt (Al) is highly similar to MIL-53 as (Al). It can be seen from Figures 2b and 2c that MIL-53 as (Al) and MIL-53 lt (Al) are consistent with the standard XRD patterns, which shows that MIL-53(Al) was successfully obtained. The XRD patterns showed that the diffraction peak of (110) appeared at 2θ = 9.5°, while the diffraction peaks of (211) and (220) overlap to form a peak at 2θ = 18.4°. These diffraction peaks are consistent with those previously reported in the literature [33,36–39].

The pore size and surface area of MIL-53(Al) were determined via the Barret–Joyner–Halenda (BJH) method and BET method, respectively. In Figure 2d, the MIL-53(Al) shows the type-IV isotherm, in which there is an H3 hysteresis loop between the adsorption–desorption isotherm. This shows that MIL-53(Al) is rich in microporous structure. Other parameters such as specific surface area and pore size are shown in Table. The BET surface area of MIL-53 as (Al) is 822.91 m^2^/g. BET surface area of MIL-53 lt (Al) is 1141.45 m^2^/g, which is close to the BET surface area of 1184 m^2^/g reported in the literature [40]. The micropore volume of MIL-53 as (Al) is only 0.2895 cm3/g, which is much less than the 0.4558 cm3/g micropore volume of the activated material. The results demonstrate that the benzene 1,4-dicarboxylic acid in the channels of MIL-53 lt (Al) has been removed.

**Figure 3:**
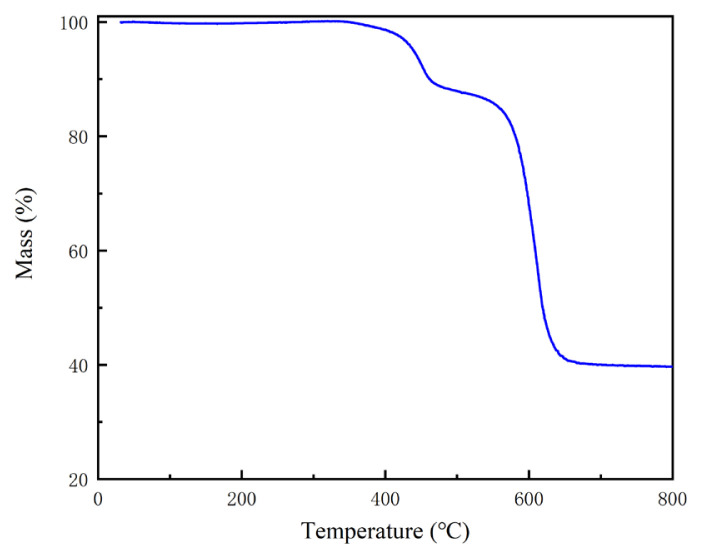
TGA curve of MIL-53 as (Al).

The TGA analysis of MIL-53 as (Al) is shown in Figure 3. Weight loss was not found before 300 °C. The first mass loss of 11.38% occurred between 346 °C and 502 °C, which was due to the loss of benzene 1,4-dicarboxylic acid in the pores. The framework began to collapse at 552 °C with a mass loss of 48.62%, and the structure has been destroyed. Unreacted benzene 1,4-dicarboxylic acid can be estimated to be 18.38%. At 700 °C, the final residue is amorphous Al_2_O_3_. From the TGA results, it can be seen that MIL-53 as (Al) can exist stably before 550 °C.

### 3.2. MIL-53 (Al) catalytic performance test

#### 3.2.1 Effect of the amount of catalyst

**Figure 4:**
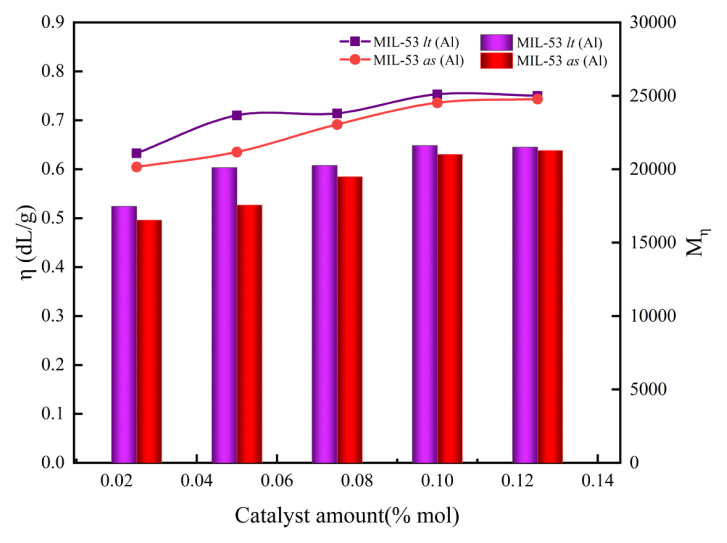
The effects of catalyst amount on the properties of PET synthesized (the ordinate of the line graph is η, and the ordinate of the bar graph is the M_η_) The molar ratio of EG to BDC is 1.3:1, polycondensation reaction temperature is 280 °C, and reaction time is 120 min.

Benzene 1,4-dicarboxylic acid and ethylene glycol (EG) in a molar ratio of EG/BDC = 1.3 were loaded in 5 L stainless-steel batch reactor equipped with a paddle agitator (50 Hz). Under the conditions of polycondensation reaction temperature of 280 °C, reaction time of 120 min and reaction pressure of 100 Pa, the catalyst content (0.025 mol%, 0.05 mol%, 0.075 mol%, 0.1 mol%, 0.15 mol%) for PET performance was studied. The research results are shown in Figure 4.

It is obvious from Figure 4 that the influence of MIL-53 (Al), before and after activation, on the intrinsic viscosity of PET has almost the same trend. As the catalyst molar ratio increases, the intrinsic viscosity of PET increases first and then gradually tends to remain unchanged. When the catalyst molar ratio is less than 0.1 mol%, the intrinsic viscosity of PET increases rapidly with the increase of its dosage. The intrinsic viscosity of PET obtained by MIL-53 as (Al) increased from 0.601 dL/g to 0.736 dL/g, and the corresponding viscosity average molecular weight increased 16,538 to 21,041 g/mol. While intrinsic viscosity of PET obtained by MIL-53 lt (Al) increased from 0.633 dL/g to 0.753 dL/g, and the corresponding viscosity average molecular weight increased 17,481 to 22,191 g/mol. When the amount of catalyst is greater than 0.1 mol%, the intrinsic viscosity and molecular weight of PET basically no longer changes. Mainly because when the catalyst is used in an amount less than 0.1 mol%, the number of active sites is the main factor affecting the rate of reaction. When the amount of catalyst reaches 0.1 mol%, the number of active sites in the reaction system tends to be saturated, and increasing the amount of catalyst will not affect the intrinsic viscosity of PET [41]. The highest intrinsic viscosity of PET obtained from MIL-53 as (Al) and MIL-53 lt (Al) is 0.744 dL/g and 0.753 dL/g, respectively and the highest molecular weights obtained are 21,285 and 22,208. It is not difficult to find from the above data that the activation effect on catalyst amount and viscosity is not significant, which indicates that the reaction is carried out on the catalyst surface. Because the average pore size of the catalyst is only 3.3 nm, it is difficult for macromolecules to enter the pores. It is noted that with the increase of the amount of catalyst, the hue of the product will be getting worse. This is because, with the amount of catalyst, the reaction rate of side reactions also increases resulting in a more colored group. In summary, it is more appropriate when the catalyst molar ratio is 0.05 mol%.

#### 3.2.2 Effect of the polycondensation time

**Figure 5:**
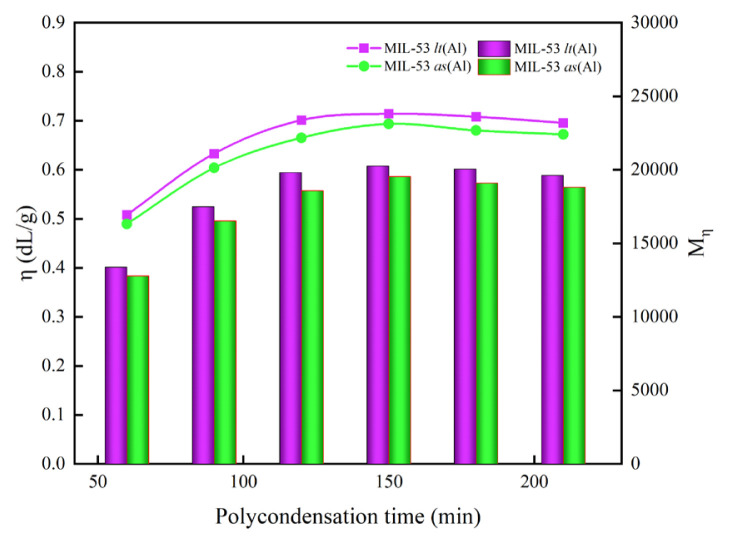
The effects of polycondensation time on the properties of PET synthesized (the ordinate of the line graph is η, and the ordinate of the bar graph is the M_η_) The molar ratio of EG to BDC is 1.3:1, polycondensation reaction temperature is 280 °C, and molar ratio of catalyst is fixed at 0.05%.

The same reaction temperature, reaction reactor, reactant ratio and reaction pressure as in 3.2.1 were used, but the molar ratio of catalyst was fixed at 0.05%. The effect of the polycondensation reaction time on the properties of PET was explored under these reaction conditions, and the results of the study are shown in Figure 5.

It can be seen from Figure 5 that the two curves have substantially the same trend. When the reaction time of the polycondensation stage is less than 150 min, the intrinsic viscosity of PET increases rapidly as the reaction time increases. The reaction time was increased from 60 min to 150 min, the intrinsic viscosity of PET catalyzed by MIL-53 as (Al) increased from 0.489 dL/g to 0.693 dL/g, and the corresponding molecular weight increased from 12,786 to 19,551. While catalyzed by MIL-53 lt (Al) increased from 0.508 dL/g to 0.714 dL/g. and the corresponding molecular weight increased from 13,380 to 20,261. The intrinsic viscosity of PET began to decrease slowly when the polycondensation time was extended from 150 min to 240 min. This trend occurs because when the reaction time is less than 150 min, in this reaction system, the polymerization rate of the reactants is greater than the rate of the side reaction, so prolonging the reaction time will make the polycondensation reaction more complete. It is not difficult to find from Figure 5 that the intrinsic viscosity of PET is the highest that the reaction time is 150 min, indicating that the polymerization reaction of PET is basically completed at 150 min. If the reaction time continues to be extended, the thermal degradation and thermal oxidative degradation rate is significantly greater than the polymerization rate [42], resulting in the intrinsic viscosity is reduced. Therefore, the reaction time of the polycondensation stage is 150 min.

#### 3.2.3 Effect of the polycondensation temperature

**Figure 6:**
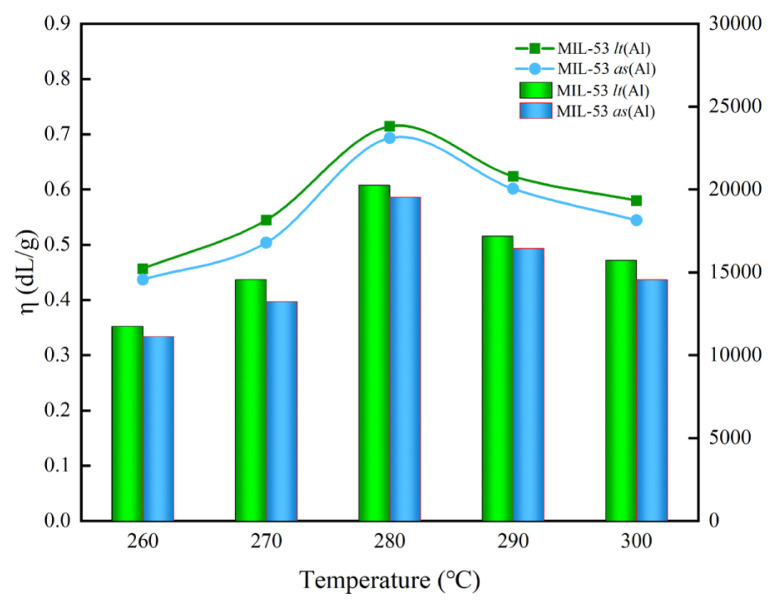
The effects of polycondensation temperature on the intrinsic viscosity of PET (the ordinate of the line graph is η, and the ordinate of the bar graph is the M_η_). The molar ratio of EG to BDC is 1.3:1, polycondensation reaction time is 120 min, and molar ratio of catalyst is 0.05%.

The same reactant ratio, reactor, and reaction pressure were used as in 3.2.1, but the molar ratio of the catalyst was fixed at 0.05% and the polycondensation reaction time was determined to be 150 min. The effect of the reaction temperature in the polycondensation stage on the intrinsic viscosity of PET was studied, and the results are shown in Figure 6.

It can be found from Figure 6 that when the reaction temperature in the polycondensation stage is lower than 280 °C, the intrinsic viscosity of PET increases rapidly as the reaction temperature increases. The reaction temperature continued to rise from 280 °C to 300 °C, and the intrinsic viscosity of PET obtained by MIL-53 lt (Al) catalyzed quickly decreased from 0.714 dL/g to 0.580 dL/g, while obtained by MIL-53 as (Al) decreased from 0.693 dL/g to 0.544 dL/g. This is mainly because the increase in the reaction temperature not only facilitates the progress of the polymerization reaction but also increases the rate of thermal degradation and thermal oxidative degradation reactions. In particular, after the reaction temperature reached 280 °C, thermal degradation and thermal oxidation degradation rate significantly higher than the polymerization rate [43], and the intrinsic viscosity of PET began to decrease rapidly. So, using MIL-53 lt (Al) and MIL-53 as (Al) as catalyst, the reaction temperatures were at 280 °C more appropriate.

### 3.3. Conceivable mechanistic consideration on MIL-53 (Al) catalysis of polycondensation reaction

**Figure 7:**
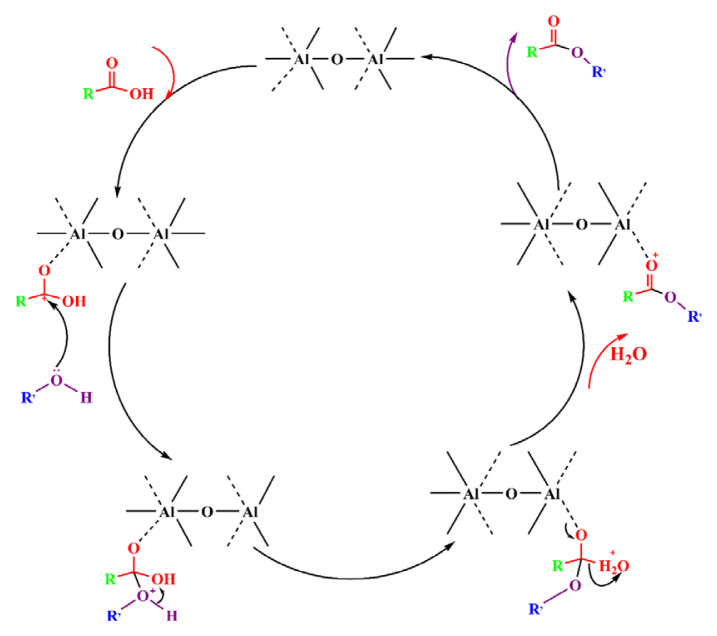
The proposed mechanism of the PET syntheses catalyzed by MIL-53.

From the above data, it can be seen that MIL-53(Al) has little effect on the intrinsic viscosity of PET before and after activation, which shows that the polymerization reaction is carried out oan the surface of the catalyst. This is because the oligomer molecules formed after the end of the esterification reaction are relatively large, and it is difficult for them to enter the pores, and the possibility of entering the pores gradually decreases as the molecular weight gradually increases as the polymerization reaction progresses. It is speculated that the reaction mechanism is based on the previous research on the polymerization reaction mechanism of PET [10,11,44]. The reaction mechanism of MIL-53 catalyzed polymerization is rationally proposed in Figure 7.

First, the carboxylic acid carbonyl is coordinated onto the active Lewis acid site of the Al oxygen cluster in MIL-53, the double bond of the carbonyl oxygen is opened, and the carbonyl is further polarized to form a carbocation. The lone-pair electron on the alcohol hydroxyl group in the reaction system will be attracted by it to undergo a nucleophilic reaction, so that the alcohol hydroxyl group is connected to the carbonyl group. Then the hydrogen atom on the alcohol hydroxyl group occurs an intramolecular proton shift, and the other hydroxyl group is removed in the form of water molecule to form a coordination ester. Finally, another carboxylic acid coordination substitution releases the resulting coordination ester to start the next catalytic cycle.

## 4. Conclusion

In summary, MIL-53 as (Al) and MIL-53 lt (Al) were used to synthesize PET for the first time, and the results showed that the catalytic performance of the two catalysts differed slightly. This is because it is more difficult for the oligomer molecules formed at the end of the esterification reaction to entering the pores, and the probability of entering the pores becomes smaller as the molecular weight increases. This proves that the reaction is carried out on the surface of the catalyst. The content of catalyst, the polycondensation reaction time and the reaction temperature are discussed. The results proved that the PET performance was optimal when the catalyst molar content was 0.05%, the reaction time was 150 min and the temperature was 280 °C. Because of its stability, MIL-53 (Al) can be added before the esterification stage and will not occur hydrolysis. The thermogravimetric results also show that its framework will not collapse before 550 °C. Moreover, the MIL-53 (Al) synthesis method is green, the price is reasonable and the ligand is BDC, which is the same as the reactant of PET without introducing other side reactions. This research provides inspiration for the use of MOF materials as polyester catalysts. Therefore, it is of great significance to develop more stable and green MOFs, as heterogeneous catalysts instead of antimony-based catalysts, to be applied to industrial production.

## Figures and Tables

**Table:** Comparison of MIL-53 before and after activation.

	BET surface area	t-Plot micropore volume	Mean pore diameter
MIL-53 as (Al)	822.91 m^2^/g	0.2895 cm^3^/g	1.8 nm
MIL-53 lt (Al)	1141.45 m^2^/g	0.4558 cm^3^/g	3.3 nm
